# Magnetic resonance cholangiopancreatography for afferent loop syndrome

**DOI:** 10.1002/ccr3.2045

**Published:** 2019-02-07

**Authors:** Kazuhide Takata, Akira Anan, Kaoru Umeda, Shotaro Sakisaka

**Affiliations:** ^1^ Department of Hepatology Hakujyuji Hospital Fukuoka Japan; ^2^ Department of Gastroenterology and Medicine Fukuoka University Faculty of Medicine Fukuoka Japan; ^3^ Department of Internal Medicine Shiida Clinic Fukuoka Japan

**Keywords:** afferent loop syndrome, magnetic resonance cholangiopancreatography

## Abstract

Afferent loop syndrome (ALS) is a rare but serious complication after gastrectomy. When a patient is diagnosed with ALS using computed tomography, magnetic resonance cholangiopancreatography may help in delineating the exact cause of ALS and determining an appropriate management.

A 73‐year‐old man who underwent Roux‐en‐Y gastrectomy for gastric cancer 5 years earlier presented to our hospital with severe epigastric pain. Contrast‐enhanced computed tomography (CT) revealed acute pancreatitis caused by afferent loop syndrome (ALS) (Figure 1). Magnetic resonance cholangiopancreatography (MRCP) revealed a dilated afferent loop and dilated pancreatic and bile ducts. Furthermore, the stenotic region showed a bird's beak sign (Figure 2).

**Figure 1:**
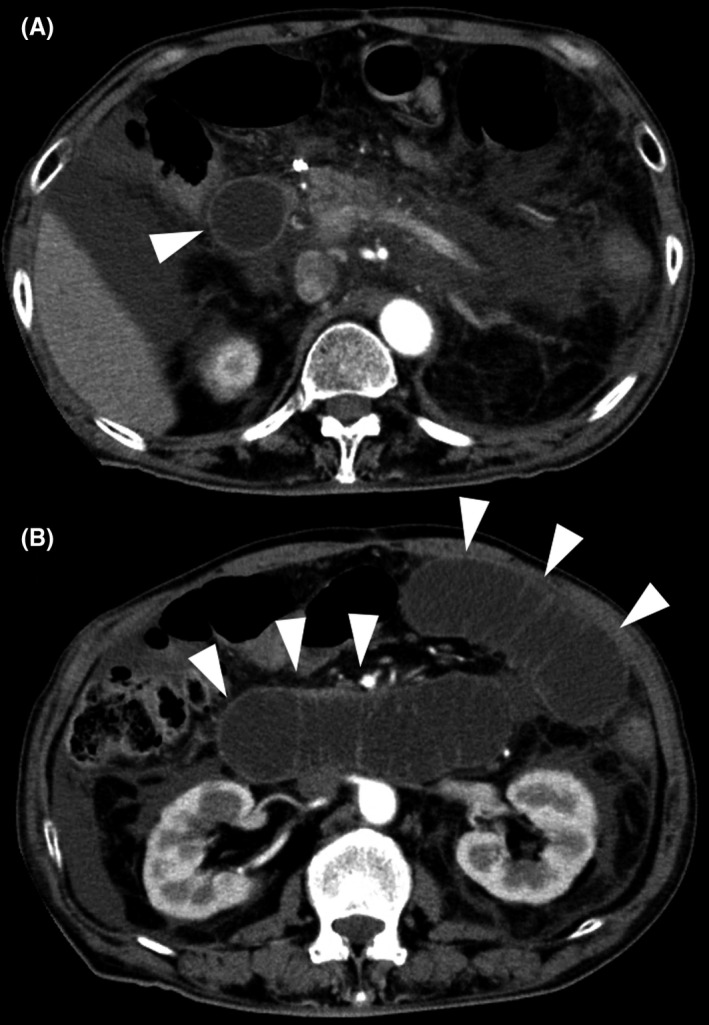
(A and B) Contrast‐enhanced computed tomography revealed swelling in the pancreas and a dilated afferent loop (arrow heads)

**Figure 2:**
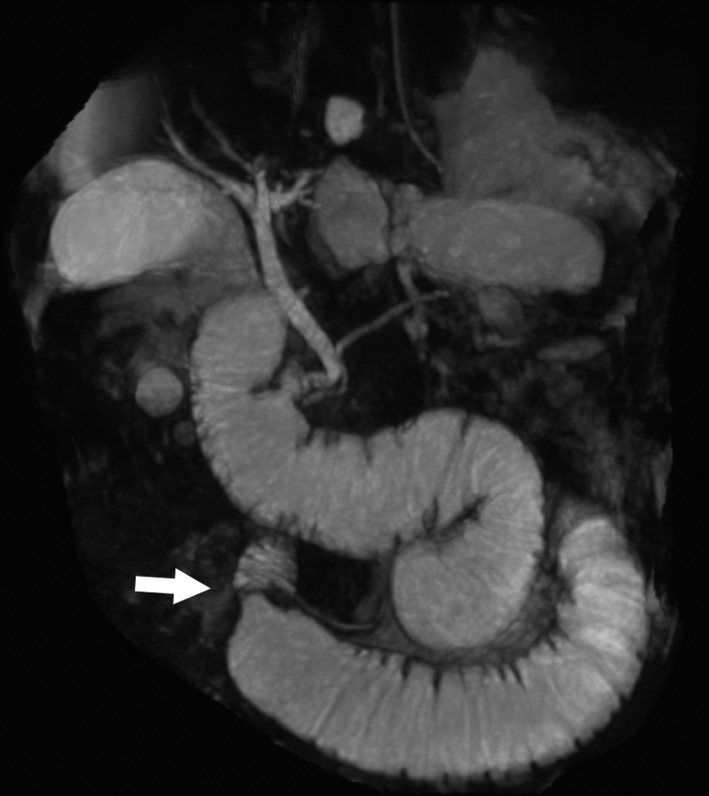
Magnetic resonance cholangiopancreatography showed the dilated afferent loop as well as the bile and pancreatic ducts. The afferent loop was obstructed on the proximal side, and the point of obstruction showed smooth tapering resembling a bird's beak (arrow)

Afferent loop syndrome is a mechanical obstruction of the afferent limb that occurs after Billroth II or Roux‐en‐Y gastrectomy. MRCP was useful in this case for three‐dimensional visualization of the obstructed afferent loop containing the bile and pancreatic ducts. In particular, MRCP showed that the afferent loop was filled with intestinal, biliary, and pancreatic fluids with no intestinal gas. Furthermore, MRCP revealed a bird's beak sign, which is the commonest sign indicating torsion. Endoscopic drainage was unsuccessful because of a difficult cannulation into the dilated afferent loop. Resection and reconstruction of the necrotic intestine resulting from an internal hernia were performed in a method similar to a previous report.^1^ The patient's postoperative course was uneventful. If a physician needs accurate anatomical information for a patient with ALS, MRCP may complement CT findings and help determine treatment strategies.

## CONFLICT OF INTEREST

None declared.

## AUTHOR CONTRIBUTION

KT: conceived and designed the study, analyzed and interpreted the data, and drafted the manuscript. AA: analyzed and interpreted the data, drafted the manuscript and made critical revision. KU: acquired the data, analyzed and interpreted the data. SS: supervised the manuscript.
